# Electric and Magnetic Properties of the Multiferroic Composites Made Based on Pb(Fe_1/2_Nb_1/2_)_1−x_Mn_x_O_3_ and the Nickel-Zinc Ferrite

**DOI:** 10.3390/ma16103785

**Published:** 2023-05-17

**Authors:** Dariusz Bochenek, Artur Chrobak, Grzegorz Ziółkowski

**Affiliations:** 1 Institute of Materials Engineering, Faculty of Science and Technology, University of Silesia in Katowice, 75 Pułku Piechoty 1a, 41-500 Chorzów, Poland; 2 Institute of Physics, Faculty of Science and Technology, University of Silesia in Katowice, 75 Pułku Piechoty 1a, 41-500 Chorzów, Poland; artur.chrobak@us.edu.pl (A.C.); grzegorz.ziolkowski@us.edu.pl (G.Z.)

**Keywords:** multiferroics, multiferroic composites, ferroelectrics, ceramic materials

## Abstract

This work presents the electrophysical properties of the multiferroic ceramic composites obtained as a result of combining both magnetic and ferroelectric material. The ferroelectric components of the composite are materials with the following chemical formulas: PbFe_0.5_Nb_0.5_O_3_ (PFN), Pb(Fe_0.495_Nb_0.495_Mn_0.01_)O_3_ (PFNM_1_), and Pb(Fe_0.49_Nb_0.49_Mn_0.02_)O_3_ (PFNM_2_), while the magnetic component of the composite is the nickel-zinc ferrite (Ni_0.64_Zn_0.36_Fe_2_O_4_ marked as F). The crystal structure, microstructure, DC electric conductivity, and ferroelectric, dielectric, magnetic, and piezoelectric properties of the multiferroic composites are performed. The conducted tests confirm that the composite samples have good dielectric and magnetic properties at room temperature. Multiferroic ceramic composites have a two-phase crystal structure (ferroelectric from a tetragonal system and magnetic from a spinel structure) without a foreign phase. Composites with an admixture of manganese have a better set of functional parameters. The manganese admixture increases the microstructure’s homogeneity, improves the magnetic properties, and reduces the electrical conductivity of composite samples. On the other hand, in the case of electric permittivity, a decrease in the maximum values of *ε_m_* is observed with an increase in the amount of manganese in the ferroelectric component of composite compositions. However, the dielectric dispersion at high temperatures (associated with high conductivity) disappears.

## 1. Introduction

In material engineering, a material is generally considered functional if it possesses a physical property usable in applications [1]. Multiferroics are defined as materials that combine two or more primary ferroic order parameters (e.g., ferromagnetic, ferroelectric, ferroelastic, or ferrotoroic state) simultaneously in the same phase [2,3,4,5,6]. Generally, the known multiferroics can be classified into three classes depending on the microscopic mechanism of ferroelectricity, namely (i) hybridization effects, (ii) geometric constraints, and (iii) electronic degrees of freedom [1]. In the first multiferroic (i) class, the magnetic and ferroelectric ordering is associated with two chemically different cations [7]. This type of material may have suitable magnetic and ferroelectric properties, but their coupling is not always strong. In geometric multiferroics (second class), the ferroelectric instability has its origin in the topology of the chemical structure, and the ferroelectric distortions are driven by ionic size effects (geometric ferroelectrics feature no significant hybridization effects or significant charge transfer between cations and anions) [1]. In correlation-induced ferroelectrics (third class), the ferroelectric polarization results from the long-range order of an electronic degree of freedom such as orbital, charge, or spin. Ferroelectricity in these materials arises from a magnetic spiral structure. Magnetic order and ferroelectricity are directly coupled since polarization does not occur without magnetic order [1]. The perovskite manganites also have many interesting properties [8]. Specifically, in the rare-earth manganites *R*MnO_3_ (where *R* represents trivalent rare-earth elements), the transition metal ions and their oxidation states are the main factor to control their multifunctional properties in magnetism and electricity [9].

The particular interest in multiferroic materials is mainly because they make it possible to exploit the functionalities of both ferroic orders [10]. Multiferroics may be advantageous to apply faster, smaller, more energy-efficient data-storage technologies [7]. For example, an electric bit to establish a four-state memory element may complement a magnetic bit in the memory application. Coupling between the ferromagnetic and the ferroelectric states might induce novel functionalities not present in either state alone [7,11,12]. If magnetoelectric coupling is present, device applications could be realized where information is written magnetically but stored in the electric polarization [13]. Future applications could also exploit toroidal moments and optical effects that arise from the simultaneous breaking of time-reversal and space-inversion symmetries [14,15].

Obtaining a multiferroic material with high multifunctional properties (where the different ferroic properties are united within one material) is difficult. In the case of the oxidic perovskites with the chemical formula ABO_3_, obtaining magnetic properties is limited because magnetism becomes established via transition metal ions such as Ni^2+^, Fe^3+^, and Mn^4+^ (with partially filled d shells). On the other hand, ferroelectric perovskite materials contain transition metal ions with empty d shells, e.g., Zr^3+^, Ti^4+^, Nb^5+^, Ta^5+^, W^6+^ [6]. They are conducive to non-centrosymmetry due to their ability to form covalent bonds with neighboring oxygen ions. Consequently, real d electrons, limiting multiferroicity, restrict the process, i.e., the coexistence of magnetic and electric long-range order [16]. In [17], the author points out the incompatibility of magnetic and electric order in perovskite materials, and ways to circumvent this conflict are analyzed.

To provide new magnetoelectric coupling mechanisms, in many works, various solutions for connecting magnetic and ferroelectric components in composite materials are used, e.g., in the form of bulk composites [18,19,20,21,22], in a multi-layered form [23], ferromagnetic-multiferroic heterostructures [24,25], and thin films [26,27,28]. The composite-type multiferroics, containing both ferroelectric and magnetic phases, can yield a high magnetoelectric (ME) coupling even though neither the ferroelectric (piezoelectric) phase nor the magnetic phase shows the ME effect [1]. The connection of a magnetic material with a ferroelectric is used in the design of multiferroic composite materials [18,19,20]. One of the interesting ferroelectric materials in terms of application is Pb(Fe_0.5_Nb_0.5_)O_3_ (PFN). PFN is a perovskite material in which, in general, the chemical formula (ABO_3_) lead ions are located in positions A, while iron and niobium ions are located in octahedral positions B (alternatingly) [29,30,31,32]. PFN shows two ordered antiferromagnetic and ferroelectric subsystems (the magnetic phase transition takes place at about −130 °C temperature, whereas the ferroelectric phase transition occurs at about 110 °C temperature) [33,34]. PFN is used as a dielectric and magnetic medium for multi-layer ceramic condensers and coils, multi-layer ceramic capacitors (MLCC) and inductors, multi-layer microwave resonators and filters as tunable transducers, electrostriction actuators, servomotors, microcontrollers, sensors, detectors, and memory devices [35,36,37,38,39,40,41,42]. In order to improve the physical properties of PFN, doping of the basic composition is most often used [43,44]. Previous studies have shown that the admixture of manganese results in ordering the microstructure, which positively affects the properties of the PFN multiferroic material [45,46].

As a magnetic component in multiferroic composites, it uses ferrites with various properties, including cobalt [47], cobalt-zinc [20], nickel-zinc [48], and manganese-zinc [49]. One of the undesirable effects of introducing a magnetic element (ferrite) affecting the properties of multiferroic ceramic composites is a significant increase in electrical conductivity [50]. This work aims to obtain multiferroic ceramic composites with good dielectric and magnetic properties at room temperature having an appropriate microstructure—favorable for the possibility of applying a high electric field (in the process of polarization of composite samples). Since one of the ways to achieve the above goal is the doping of the basic composition, an admixture of manganese is introduced into the PFN, which, added in appropriate amounts, organizes the microstructure of perovskite ceramic materials (increases uniformity of grain size) [51]. The paper presents the results of magnetic and electric studies of multiferroic composites obtained based on a ferroelectric-ferromagnetic PbFe_0.5_Nb_0.5_O_3_ powder, both undoped and doped with MnO_2_ manganese oxide (in the amount of 1.0 and 2.0 at.%), as well as nickel-zinc ferrite Ni_0.64_Zn_0.36_Fe_2_O_4_ (NZF) with the magnetic properties and sufficiently high resistance at room temperature. The ferroelectric and ferrite phase content in the multiferroic composites was 90/10.

## 2. Experiment

The technological process included three main stages, i.e., obtainment of the PbFe_0.5_Nb_0.5_O_3_ (PFN) and Pb(Fe_0.5_Nb_0.5_)_1−x_Mn_x_O_3_ (PFNM) powders (stage 1), obtainment of the Ni_0.64_Zn_0.36_Fe_2_O_4_ (NZF) ferrite powder (stage 2), and obtainment of the composite ceramic samples (stage 3). In the first stage, the input components in the technological process of the ceramic powder were simple oxides: PbO (99.99%, POCH, Gliwice, Poland), Fe_2_O_3_ (99.9%, POCH, Gliwice, Poland), Nb_2_O_5_ (99.9%, Sigma-Aldrich, St. Louis, MO, USA), and MnO_2_ (99%, Sigma-Aldrich, St. Louis, MO, USA), which were weighed in stoichiometric proportions (in the case of PbO, an excess powder was applied). The input powder mixture was milled for 12 h in the ball mill (Fritsch Pulverisette 6, Idar-Oberstein, Germany), and next, the mixture was calcined at 800 °C for 3 h. In the second stage, the nickel-zinc ferrite (Ni_0.64_Zn_0.36_Fe_2_O_4_) powder was obtained from simple oxides (99.99%), i.e., ZnO, Fe_2_O_3_ and NiO (POCH, Gliwice, Poland), which, after milling (as above), were synthesized under the conditions of 1100 °C/4 h. In the third part of the technological process, the ferroelectric and magnetic powder have been linked together. The ceramic powders were weighed in the proportion of 90/10 (ferroelectric and magnetic phase, respectively) and next mixed in a ball mill (Fritsch Pulverisette 6) for 8 h (in ethanol). The multiferroic composite powder was calcined at 900 °C/4 h while final sintering was conducted via free sintering method (pressureless) at 1150 °C for 4 h. In the last stage of the technological process, the samples were sanded down, polished, and annealed, and on both surfaces of the composite samples, silver electrodes were placed. Composite samples were marked as follows: (i) 0.9[PbFe_0.5_Nb_0.5_O_3_]-0.1[Ni_0.64_Zn_0.36_Fe_2_O_4_] (PFN-F), (ii) 0.9[Pb(Fe_0.495_Nb_0.495_Mn_0.01_)O_3_-0.1[Ni_0.64_Zn_0.36_Fe_2_O_4_] (PFNM_1_-F), and (iii) 0.9[Pb(Fe_0.49_Nb_0.49_Mn_0.02_)O_3_]-0.1[Ni_0.64_Zn_0.36_Fe_2_O_4_] (PFNM_2_-F).

X-ray tests were conducted on a diffractometer Phillips X’Pert (Panalytical, Eindhoven, the Netherlands) at room temperature (*RT*) and in the 2*θ* angle range from 15° to 62° (CuK_α_ = 1.54056 Å radiations). For the study of the microstructure of the composite samples, the scanning electron microscope Jeol JSM-7100F TTL LV (Jeol Ltd., Tokyo, Japan) was used. Two types of imaging capture were used, namely the SB manner (signals from secondary and backscattered electrons) and the BSE (detection of backscattered electrons). The chemical composition analysis of multiferroic composites (using spot, linear, and surface analyses) were performed by energy dispersive spectrometer EDS (JSM-7100F TTL LV Jeol Ltd., Tokyo, Japan). The mapping of the surfaces, i.e., electron probe microanalysis EPMA, was also performed using the EDS detector. The average grain size was performed in the ImageJ program. Relative density of the composite samples was estimated according to the Archimedes method. Dielectric properties of the composite samples were tested on the QuadTech 1920 Precision LCR meter (Maynard, MA, USA) from *RT* to 300 °C and frequencies 0.2–100 kHz. DC electric conductivity studies were performed by a digit multimeter Keysight 34465A (Santa Rosa, CA, USA) from *RT* to 300 °C. Ferroelectric properties (*P*–*E* loop) were tested on the Sawyer-Tower circuit with the help of high-voltage amplifier Matsusada Inc. HEOPS-5B6 precision (Kusatsu, Japan) at *RT* and 5 Hz. The data were captured using an A/D, D/A transducer card (National Instrumental, Austin, TX, USA), and the LabView computer program. Magnetic properties were obtained by applying the Quantum Design PPMS system (PPMS 7T ACMS module, San Diego, CA, USA), within a temperature range from −268 °C to 30 °C.

The composite samples were subjected to poling in silicon oil using a high voltage power amplifier Matsusada Inc. HEOPS-5B6 precision (Kusatsu, Japan) in the following poling conditions: field *E_pol_* = 10 kV/cm, time *t_pol_* = 1 h, and temperature *T_pol_* = 100 °C (cooling to *RT* was carried out with the application of an electric field). The piezoelectric resonance measurements of polarized samples were carried out using QuadTech 1920 Precision LCR Meter (Maynard, MA, USA) over a broad frequency range (100 Hz–1 MHz) at *RT*, while the piezoelectric parameters were calculated through the resonance and anti-resonance method. The piezoelectric coefficient *d*_33_ was measured at *RT* using a YE2730A d33 meter (APC International Ltd., Mackeyville, PA, USA).

## 3. Results and Discussion

### 3.1. Crystal Structure

**Figure 1:**
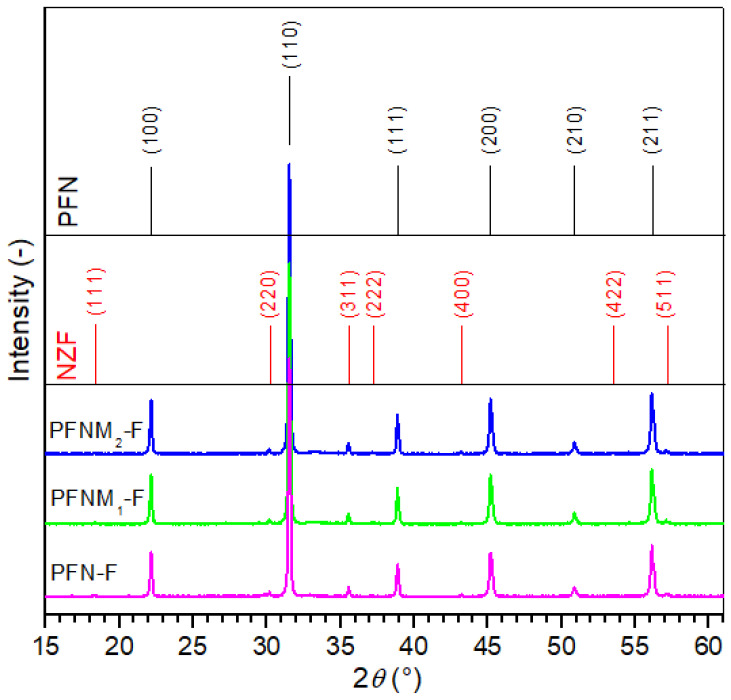
X-ray diffraction patterns for the multiferroic composites.

The X-ray analysis (Figure 1) showed the presence of two main phases in the diffraction patterns, originating both from the PFN-type material (firm peaks) and the NZF ferrite (with much lower intensity). In the case of PFN, the analysis confirmed that it belongs to the perovskite structure of the tetragonal system with the P4*mm* space group. A good match of the experimental results of the PFN material was obtained with the pattern no. card 04-009-5124. The admixture of manganese in PFN in the amount of 1 and 2% does not cause a marked change in the intensity and width of the main diffraction patterns (derived from PFN). In the case of the Ni_0.64_Zn_0.36_Fe_2_O_4_ ferrite powder, X-ray diffraction patterns showed that it had a spinel structure from a regular crystal system and space group F*d*-3*m* (no. card 01-077-9718). X-ray measurements of the multiferroic composites also confirmed the absence of impurities and a foreign phase (including the undesirable pyrochlore phase).

### 3.2. Microstructure

**Figure 2:**
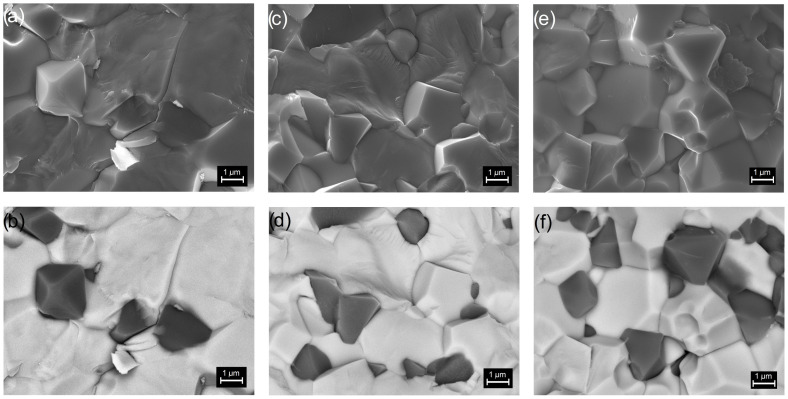
SEM images of the microstructure of the multiferroic composites: PFN-F (**a**,**b**), PFNM_1_-F (**c**,**d**), and PFNM_2_-F (**e**,**f**) (magnification ×10,000). SB technique (**a**,**c**,**e**), and BSE technique (**b**,**d**,**f**).

**Figure 3:**
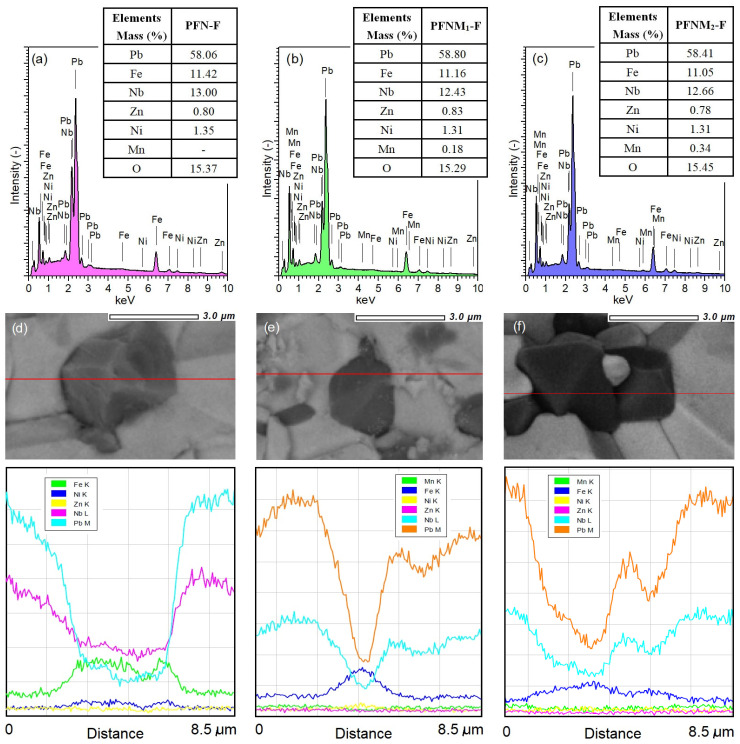
The surface EDS analysis of chemical elements of the multiferroic composites: PFN-F (**a**), PFNM_1_-F (**b**), and PFNM_2_-F (**c**) with the experimental results in the tables, and the linear EDS analysis (red line) of the PFN-F (**d**), PFNM_1_-F (**e**), and PFNM_2_-F (**f**).

**Figure 4:**
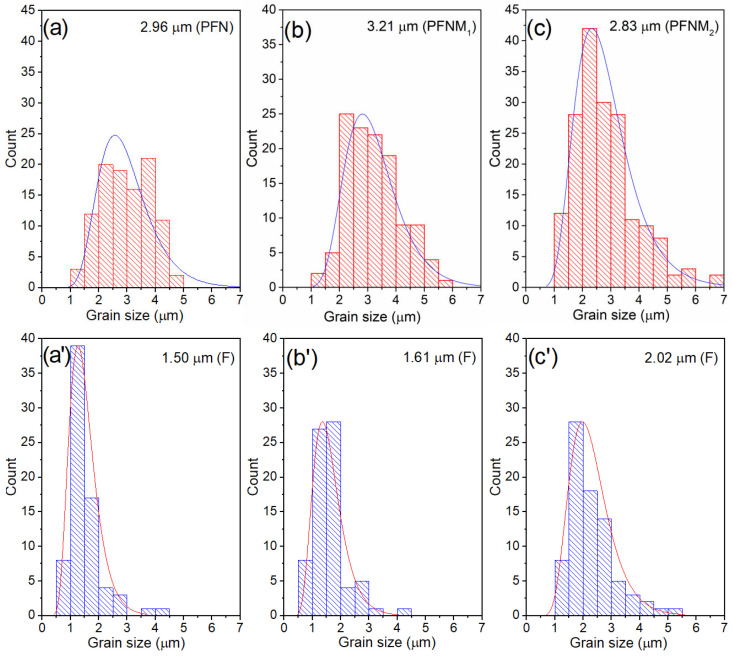
Grain size distribution for the multiferroic composites: PFN-F (**a**,**a’**), PFNM_1_-F (**b**,**b’**), and PFNM_2_-F (**c**,**c’**), ferroelectric grains red color, ferrite grains blue color.

Multiferroic composite samples are characterized by high density and low porosity (Table 1). Figure 2 presents SEM images of the cross-section surface morphology of ceramic composite samples obtained in both the SB technique (upper images) and the BSE technique (bottom images). Well-crystallized grains with the correct shape with visible sharp grain boundaries characterize the microstructure of the multiferroic composite samples. The matrix of the composite forms the grains of the ferroelectric material (bright areas in BSE images) that surround the magnetic material grains (dark areas in BSE images). This assignment was established based on the spot and linear EDS analyses performed. Figure 3d–f shows the linear EDS analyzes for selected microstructure areas of the samples, i.e., for PFN-F (d), PFNM_1_-F (e), and PFNM_2_-F (f). The waveforms for individual elements show maximum or minimum values in areas rich or poor in a given element, respectively. The magnetic component grains also show a regular shape and well-defined grain boundary edges. In the case of the pure PFN-F sample (Figure 2a,b), the grains of the ferroelectric component are much larger than in the case of other composite samples with manganese doped. The introduction of manganese to the basic PFN ferroelectric component (i.e., PFNM_1_-F, PFNM_2_-F samples) increases the homogeneity of the ferroelectric grains in composite microstructure. Figure 4 shows the grain size distribution of the multiferroic composite samples. The average ferroelectric grain size is in the range from 2.83 to 3.21 μm, while in the case of ferrite grains, it is in the range from 1.50 to 2.02 μm. The admixture of manganese introduced into PFN improves the ordering of microstructure grains, increasing their homogeneity [51].

The final properties and potential applications of multiferroic composites depend on the perfection and condition of the microstructure. Paper [52] shows that domain walls may play an important role in electronic devices due to their small size as well as the fact that their location can be controlled. It has been shown that the conductivity correlates with structurally driven changes in both the electrostatic potential and the local electronic structure. It causes a decrease in the bandgap at the domain wall. In the example of BaTiO_3_ in [14], the authors demonstrated that multiferroicity can emerge at interfaces or domain walls. It was presented that the magnetism extends over several unit-cells in the ferroelectric layer, opening the way towards artificial multiferroic tunnel barriers. This type of interface-induced multiferroics could exhibit exciting non-reciprocal optical effects and ferrotoroidic order [14].

The quality and stability of the domain structure of piezoelectric materials (i.e., stability of electro-physical parameters) at their operating temperature closely affect the working parameters of microelectronic equipment. An increase in the requirements which those materials results that work is still being conducted on improving the technologies of that type of multiferroic materials. The ceramics’ properties depend significantly on the technique of their technological process, beginning with a synthesis of input powders (an appropriate way of synthesizing) through their milling, compacting, and final sintering stages. During the technological process of the PFN materials, there are many difficulties to obtain a material with beneficial properties, e.g., a high electrical conductivity, dielectric loss, as well as tendency for the formation of a second non-ferroelectric phase (pyrochlore) which limits the use of pure PFN in practice. In the case of the PFN, a great isomorphism, characteristic of perovskite structure materials, enables the control of physical properties by selecting appropriate admixture and technological conditions [51]. The chemical composition of the PFN material is never stoichiometrically ideal since oxygen (anion) or lead (cation) vacancies are created during the technological process. The conduction type in the perovskite structure is connected with the concentration ratio of cation and anion vacancies. If in the crystal lattice, anion vacancies predominate over cation vacancies, and a perovskite exhibits electron conduction (n-type) while cation vacancies dominate; it exhibits hole conduction (p-type) [45].

The surface EDS analysis (Figure 3a–c) of the composites showed the presence of reflections from all elements that make up composite materials, i.e., lead, iron, niobium, manganese (ferroelectic one); nickel, zinc, iron (ferrite one); and no reflections from foreign elements. Tables in Figure 3 presented the averaged percentage results of elements (experimental) of the multiferroic composites. The EDS analysis averaged results for 5 measurements from randomly selected micro-areas of the sample surface.

**Figure 5:**
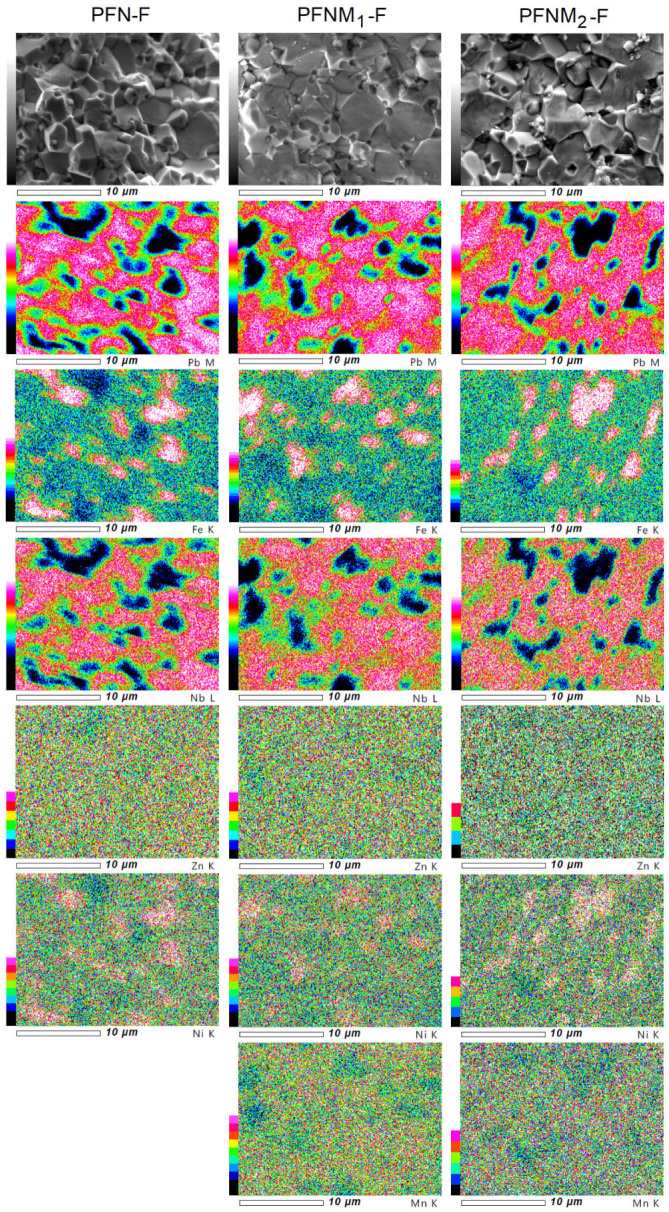
EPMA maps of the multiferroic composites: PFN-F, PFNM_1_-F, and PFNM_2_-F. For the ferroelectric component, maps of the elements lead, iron, niobium, and manganese, while for ferrite, maps of the elements nickel, zinc, and iron.

The electron probe microanalysis (EPMA) tests allow the presentation of the distribution of constituent elements in cross section of the multiferroic ceramic samples in an illustrative way. Figure 5 shows the EPMA images (heat diagram) of the individual elements distribution for multiferroic composites. In the microstructure areas of the composite samples where the ferroelectric and magnetic phases occur, the increased intensity of their components is marked. Due to the small amount of Zn and Ni elements (ferrite components) in composite compositions, the accuracy of EPMA measurement is the lowest. The EPMA test is consistent with the results of the EDS analysis (spot and linear) and with the BSE technique’s SEM images (Figure 2b,d,f).

### 3.3. DC Electric Conductivity

**Figure 6:**
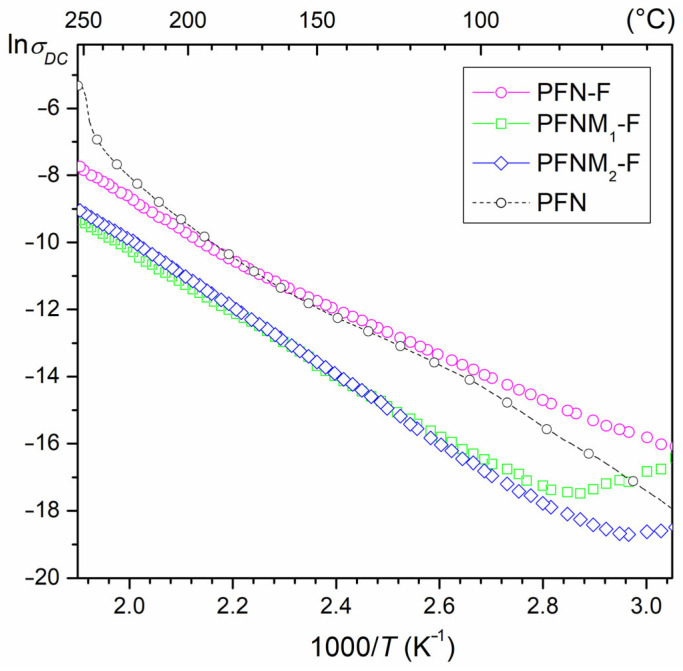
Temperature dependencies of DC electric conductivity for the multiferroic composites and PFN material.

At *RT*, the *ρ_DC_* resistivity of the composite samples has relatively high values which are 5.2 × 10^7^ Ωm (for PFN-F), 1.2 × 10^8^ Ωm (for PFNM_1_-F), and 1.8 × 10^8^ Ωm (for PFNM_2_-F). Figure 6 shows the ln*σ_DC_* (1000/*T*) plots for the three analyzed ceramic composites and PFN ceramic material. It is well known that depending on the technology used, the PFN material may exhibit high electrical conductivity and dielectric loss [53,54,55]. Obtaining a multiferroic composite of PFN and ferrite (in the proportion of 90/10) allows for reducing the electrical conductivity. With increasing temperature, the DC electrical conductivity of composite samples increases consistently, which is related to the increased drift mobility of thermally activated charge carriers [56]. The increase in electrical conductivity at higher temperatures may be ascribed to the hopping nature of charges in Fe^3+^ ↔ Fe^2+^ and Nb^5+^ ↔ Nb^4+^ [57]. It was noticed that the composite sample without Mn admixture (PFN-F) shows the highest electrical conductivity. In the case of PFN doped with Mn^4+^, taking the value of the ionic radius (*R*_Mn_ = 52 pm) into account, the manganese Mn will substitute in positions B, iron Fe (*R*_Fe_ = 67 pm), or niobium Nb (*R*_Nb_ = 69 pm). The introduction of Mn^4+^ to the main PFN composition triggers the occurrence of two cases that may result in an appropriate compensation of their effects. In the first case, an atom with a lower oxidation degree than Nb^5+^ generates acceptor centers. Holes form to cause the material to become a p-type semiconductor. In the second case, substituting the Mn^4+^ ions in the Fe^3+^ positions forms donor centers with excess electrons. Such a type of admixing causes compensation of electric charges that leads to decreased electrical conduction of the PFNM-F composite materials [46].

The temperature dependence of DC electrical conductivity obeys the Arrhenius law (1) very well. The activation energies *E_a_* of the composite samples were calculated based on the slope of the ln*σ_DC_* (1000/*T*) curves and the Arrhenius equation.

(1) $$\sigma_{DC}=\sigma_{0}\exp\left(\frac{E_{a}}{k_{B}T}\right),$$

where *σ*_0_*—*pre-exponential factor, *k_B_—*Boltzmann’s constant, *T—*absolute temperature, *E_a_—*the activation energy [58]. The activation energies *E_a_* of the composite samples are 0.58 eV (for PFN-F), 0.87 eV (for PFNM_1_-F), and 0.80 eV (for PFNM_2_-F). In the ferrites, conductivity is connected with the hopping of charge carriers between the iron ions in different valence states [59,60], whereas in the perovskite materials, it is connected among others with presence of oxygen and lead vacancies and defect dipolar effects [61,62]. The calculated *E_a_* values mainly indicate conductivity dominated by the presence of oxygen vacancies in the multiferroic composite samples [61].

### 3.4. Dielectric Properties

**Figure 7:**
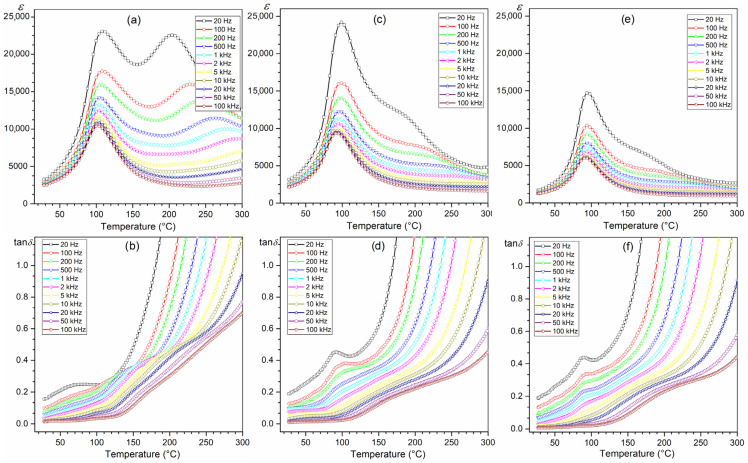
Temperature dependencies of permittivity (**a**,**c**,**e**) and dielectric loss factor (**b**,**d**,**f**) for the multiferroic composites: PFN-F (**a**,**b**), PFNM_1_-F (**c**,**d**), and PFNM_2_-F (**e**,**f**).

**Figure 8:**
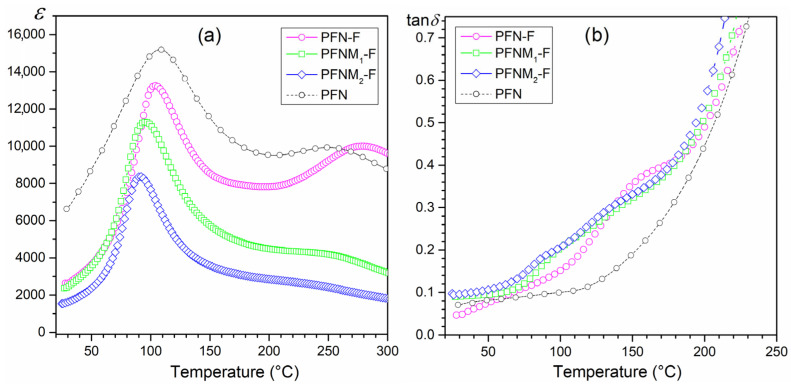
Temperature dependencies of dielectric properties for the multiferroic composites (1 kHz): permittivity (**a**) and the dielectric loss factor (**b**).

Figure 7 shows the temperature dependencies of the dielectric properties of ceramic composites, i.e., the permittivity and the dielectric loss factor (tan*δ*). All obtained composite samples have high permittivity values. At *RT*, the values of permittivity are 2750 for PFN-F, 2350 for PFNM_1_-F, and 1530 for PFNM_2_-F. Figure 8 also presents the results of permittivity and dielectric loss factor for the PFN material obtained with the classical technology for comparative purposes (1 kHz). The introduction of ferrite (in the amount of 10%) causes a decrease in permittivity values (dotted line in Figure 8). The composite PFN-F sample has the highest permittivity values in the entire measuring range and the lowest tan*δ* values at *RT*. Doping the ferroelectric component of the PFN with manganese reduces the maximum values of the permittivity, but the tan*δ* values remain at a low level (Figure 8). Multiferroic composite samples have relatively low dielectric loss factor values up to about 120 °C (Figure 8b). At room temperature, the tan*δ* values are 0.065 for PFN-F, 0.089 for PFNM_1_-F, and 0.097 for PFNM_2_-F, respectively. Above 120 °C temperature, their rapid increase is observed, which is related to the increased electrical conductivity of composite samples at high temperatures. At the same time, the phase transition from ferroelectric to paraelectric phase takes place at lower temperatures (the *T_m_* temperature gets smaller), comparing the composite sample with pure PFN (Table 1).

Moreover, for PFN-F, on the temperature courses *ε*(*T*) above the phase transition temperature, there is a local maximum of permittivity (occurring over a wide temperature range), marked by a distinct frequency dispersion (dielectric relaxation). The local maximum shifts to the higher temperature side with increasing frequency. It is commonly known that the dielectric relaxation is related to the delay in the frequency response of dielectric dipoles in an external alternating field in a time interval. The dielectric dispersion at high temperatures is related to the conductivity mechanism, i.e., space charge mechanisms of relaxation phenomena (oxygen vacancies and related defects). At high sintering temperatures, due to the volatility of PbO, lead vacancies appear [63]. In turn, easily formed lead vacancies due to charge neutrality restrictions facilitate the formation of oxygen vacancies [64]. The created conditions favor the formation of conducting electrons in the process of ionization of oxygen vacancies according to the relationships Vo ↔ Vȯ + e′ and Vȯ ↔ Vö + e′ (where Vȯ, Vö are single and doubly ionized oxygen vacancies). In the case of lead-type materials, the ionized oxygen vacancies are weakly bonded to Pb^2+^ ions, which can be described by the Kroger–Vink notation as shown in (2):

(2) $${\mathrm{Pb}}_{\mathrm{Pb}}^{x}+O_{x}\to V_{\mathrm{Pb}}^{\prime \prime }+V_{O}^{..}+\mathrm{PbO}↑$$

In the case of compositions doped with manganese (PMFM_1_-F and PMFM_2_-F), the above-mentioned phenomenon disappears (Figure 7a,c,e). It proves the lower electrical conductivity of the doped PFNM-F compositions, in which the admixture of manganese Mn^4+^ partially compensates for the emerging oxygen vacancies. This is also confirmed by the conducted studies of DC electrical conductivity, which show much lower electrical conductivity for compositions with manganese (Figure 6). Favorable results of the doping of PFN with manganese are also presented in [46,65].

Studies of the dielectric properties of multiferroic composite materials have shown that in the case of a ferroelectric composite matrix (in the form of PFN and PFNM material), the negative impact of ferrite on dielectric parameters is not as large as in many works on other composite compositions [66,67]. The negative impact of ferrite in composites is most often revealed by increased dielectric loss, reduction of the permittivity value, and blurring of the phase transition (ferroelectric/paraelectric) [49,68,69].

### 3.5. Ferroelectric Properties

**Figure 9:**
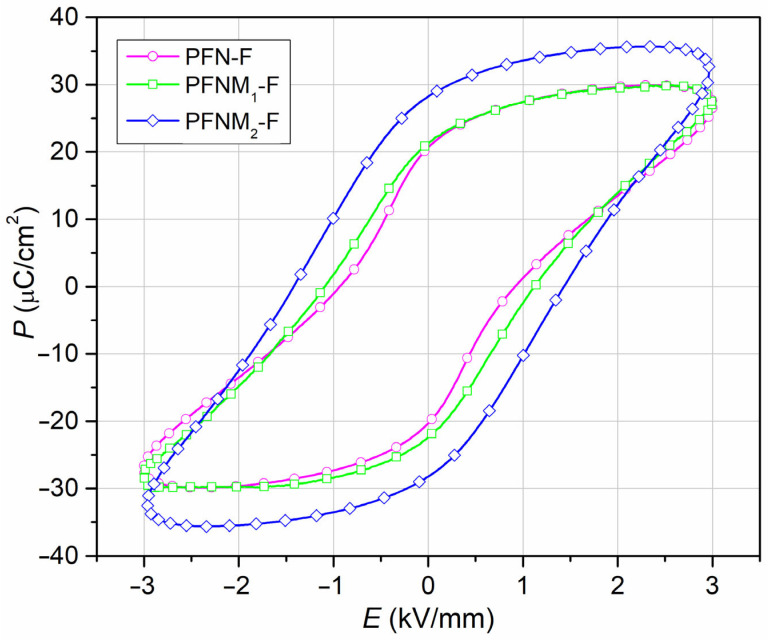
*P–E* hysteresis loop for multiferroic composites: PFN-F (a), PFNM_1_-F (b), and PFNM_2_-F (c).

Figure 9 shows ferroelectric *P*–*E* hysteresis loops for composite samples at *RT* (a frequency 5 Hz). *P*–*E* loops have large *E_c_* coercive field values and do not show saturation at a field strength of 3 kV/mm (the magnetic component of the composite much weakens the ferroelectric properties). In the case of the PFN-F, the *P–E* loop is wide (high ferroelectric hardness), while for PFNM_1_-F and PFNM_2_-F samples, the *P–E* loops are narrower (medium ferroelectric hardness). The *E_c_* values are 0.92 kV/mm for PFN-F, 1.11 kV/mm for PFNM_1_-F, and 1.43 kV/mm for PFNM_2_-F. For the external electric field applied (3 kV/mm), the *P_r_* residual polarization of ceramic composites is relatively high, and the amounts are 20.63 µC/cm^2^, 21.25 µC/cm^2^, and 28.24 µC/cm^2^ for PFN-F, PFNM_1_-F, and PFNM_2_-F, respectively. The magnetic component of the multiferroic composite samples limits the possibility of proper saturation of the *P*–*E* hysteresis loop. For a perovskite ceramic material, the *E_c_* coercive field values inform about grains size in the microstructure, i.e., a low *E_c_* value corresponds to large grains in the microstructure. In contrast, a high *E_c_* value corresponds to small grains. This regularity is confirmed by the results of the SEM microstructural research (Figure 2), considering the ferroelectric matrix grains. The lack of saturation of the *P–E* loop was also presented in [20] for cobalt-nickel composites but with much lower polarization values.

### 3.6. Magnetic Properties

**Figure 10:**
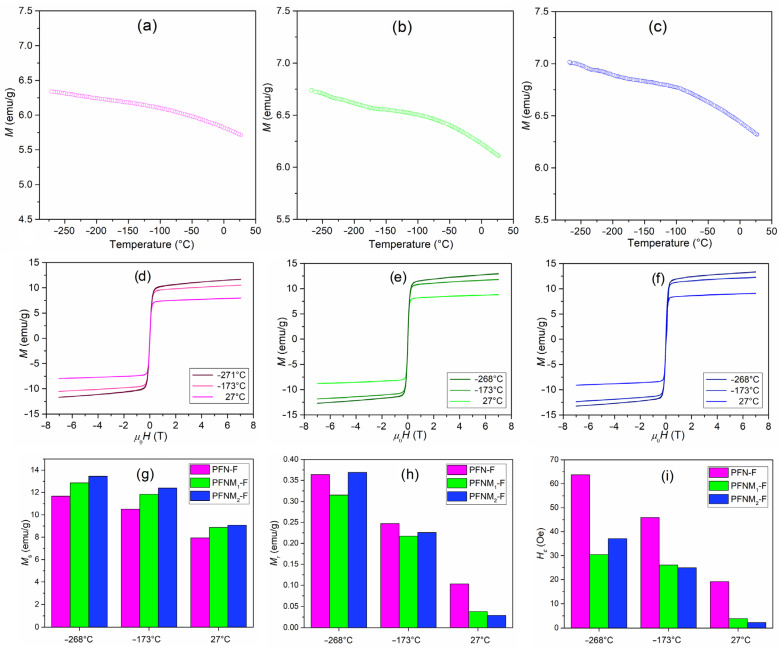
Temperature dependencies of magnetization for multiferroic composite samples: PFN-F (**a**), PFNM_1_-F (**b**), and PFNM_2_-F (**c**), magnetic hysteresis loops at –268 °C, –173 °C and 27 °C, for PFN-F (**d**), PFNM_1_-F (**e**), and PFNM_2_-F (**f**), respectively, and values form *M–H* loops: *M_s_* saturation magnetization (**g**), *M_r_* remnant magnetization (**h**), and *H_c_* coercive field (**i**).

The temperature dependence of *M* magnetization in the magnetic field of the multiferroic composites is depicted in Figure 10. The research was carried out in the temperature range from –268 °C to 30 °C and for magnetic field 0.1 T. Magnetic tests of the PFN-F composite showed the highest values of magnetization at the lowest measurement temperatures, and at higher temperatures, the magnetization values were consistently reduced. The temperature course of changes of magnetization *M*(*T*) showed a linear decrease of magnetization in a temperature range. It is a typical trend for multiferroic composite materials composed of a magnetic and ferroelectric element. The temperature magnetic tests present a strong signal coming from the ferrimagnetic phase and a weak one coming from the paramagnetic phase [70]. The admixture of manganese introduced to the main composition of PFN (a ferroelectric component of the composite) increases the magnetization values. At temperatures of –268 °C and *RT*, the highest magnetization values show the PFNM_2_-F sample, i.e., 7.02 emu/g and 6.32 emu/g, respectively. In this case, the decrease in the value of *M* is 9.97% of its initial value. In the case of the PFN-F and PFNM_1_-F samples, this parameter is equally high and amounts to 9.84% and 9.35%, respectively. In the case of PFNM_1_-F and PFNM_2_-F composite samples (with Mn admixture), the higher value of magnetization occurs due to the double exchange interaction arising from the presence of Mn^2+^/Mn^3+^ [71,72]:

(3) $${\mathrm{Mn}}^{2+}+e_{g}^{2}↔{\mathrm{Mn}}^{3+}.$$

The hopping of doubly degenerate *e_g_*^2^ electron induces the magnetic transition. In this type of material, it is possible to obtain an enhancement in magnetoelectric properties due to structural disordering originating from the tilting of MnO_6_ octahedra [73].

The magnetic hysteresis loops for all composite samples are depicted in Figure 10d–f. The figure shows *M–H* loops for temperatures –268 °C, –173 °C, and 27 °C. The composite ceramic samples show narrow magnetic hysteresis loops characteristic of the composite materials designed based on soft ferrimagnetic ferrite [74]. Generally, the examined composites are magnetically soft with a small trace of magnetic anisotropy, causing the appearance of relatively low coercivity and remanence. The anisotropy can be attributed to surface/shape effects of the magnetic grains, which almost vanish at higher temperatures by thermal energy allowing spontaneous rotation of magnetic moments. In fact, as the temperature increases, the values of *M_s_* saturation magnetization, *M_r_* remnant magnetization, and *H_c_* coercive field decrease (Figure 10g–i). For all samples, the *M–H* loops have similar shapes but differ in the magnetization values (the highest *M_s_* is for PFNM_2_-F).

### 3.7. Piezoelectric Properties

Piezoelectric parameters were examined after the poling process of the composite samples. Since the presence of ferrite in composite samples is associated with increases in electrical conductivity, the polarization process is complicated. A low polarizing field (10 kV/cm) was applied to the samples at 100 °C. Composite compositions with an admixture of manganese show higher values of piezoelectric parameters in comparison with undoped PFN. The electrical coupling coefficient for PFN-F is 0.28, while for manganese-doped compositions, it is 0.33 and 0.34, respectively, for PFNM_1_-F and PFNM_2_-F samples. Additionally, the piezoelectric module *d*_31_ has higher values, i.e., 45 pC/N (for PFNM_1_-F) and 42 pC/N (PFNM_2_-F), compared to PFN-F (29 pC/N). The tests of the *d*_33_ piezoelectric module for the analyzed set of samples show similar trends as piezoelectric parameters calculated by the resonance-antiresonance method. They are 53 pC/N, 78 pC/N, and 67, for PFN-F, PFNM_1_-F, and PFNM_2_-F, respectively.

## 4. Conclusions

The paper obtained three multiferroic composites based on ferroelectric and magnetic materials. In the composite compositions, PbFe_0.5_Nb_0.5_O_3_ (PFN), Pb(Fe_0.495_Nb_0.495_Mn_0.01_)O_3_ (PFNM_1_), and Pb(Fe_0.49_Nb_0.49_Mn_0.02_)O_3_ (PFNM_2_) were the ferroelectric components while the nickel-zinc ferrite Ni_0.64_Zn_0.36_Fe_2_O_4_ (F) was the magnetic component.

Multiferroic ceramic composites at room temperature have a two-phase crystal structure (ferroelectric from a tetragonal system and magnetic from a spinel structure) without a foreign phase. The conducted magnetic and dielectric measurements confirmed that at *RT*, the composite samples have suitable dielectric and magnetic properties. Composites with an admixture of manganese have a better set of functional parameters despite lower permittivity values. The manganese admixture introduced to the PFN (in a small amount) increases the homogeneity of the microstructure and improves the magnetic properties, as well as reducing the DC electrical conductivity of the composite samples. Improving the microstructure of the multiferroic composites also ensures effective polarization of the samples (possibility of applying a higher electric field in the polarization process) obtaining higher piezoelectric properties.

The multiferroic ceramic composites obtained by combining Mn-doped PFN (as well as PFN) and nickel-zinc ferrite show promising properties for applications in modern microelectronics and micromechatronics.

## Figures and Tables

**Table 1:** Electrophysical parameters of the multiferroic composite samples: PFN-F, PFNM_1_-F, and PFNM_2_-F.

	PFN-F	PFNM_1_-F	PFNM_2_-F
*ρ* (g/cm^3^)	7.62	7.64	7.66
*ρ_DC_* at *RT* (Ωm)	5.2 × 10^7^	1.2 × 10^8^	1.8 × 10^8^
*M_S_* (Am^2^/kg) at −268 °C	6.34	6.74	7.02
*M_S_* (Am^2^/kg) ^1^	5.71	6.01	6.32
*M*_max_	7.92	8.85	9.03
*ε* ^1^	2750	2350	1530
*T_m_* (°C)	99	96	92
*ε_m_*	18,570	11,220	8320
tan*δ* ^1^	0.065	0.089	0.097
tan*δ* at *T_m_*	0.077	0.195	0.191
*E_a_* (eV)	0.58	0.87	0.80
*P_r_* (µC/cm^2^) ^1,2^	20.63	21.25	28.24
*E_c_* (kV/mm) ^1,2^	0.92	1.11	1.43
*d*_33_ (pC/N) ^1^	53	78	67
*k_p_* ^1^	0.28	0.33	0.34
*d*_31_ (pC/N) ^1^	29	45	42
*Q_m_* ^1^	427	123	72

^1^ test at *RT*, ^2^ test for 5 Hz and *E* = 30 kV/cm.

## Data Availability

Data are contained within the article.
